# Advancing antifungal therapy: exploring targeted CFW-PEc-enhanced ethosomal formulations of amphotericin B against cryptococcal pneumonia

**DOI:** 10.1128/spectrum.01729-25

**Published:** 2025-09-25

**Authors:** Guoting Shi, Mengshun Li, Lili Chu, Baocheng Tian, Mengxin Li, Haiyan Wang, Huihui Zhou, Yanchun Han, Chunxiao Meng, Chen Ding, Sixiang Sai

**Affiliations:** 1 School of Pharmacy, Binzhou Medical University 74705, Yantai, Shandong, China; 2 Department of pathology, Affiliated Yuhuangding Hospital of Qingdao University, Yantai, Shandong, China; 3 College of Life and Health Science, Northeastern University 1848, Shenyang, China; Debreceni Egyetem, Debrecen, Hungary

**Keywords:** CFW-PEc-AmB-ethosomes, antifungal activity, drug delivery systems, *Cryptococcus neoformans*

## Abstract

*Cryptococcus neoformans* is a significant and pathogenic encapsulated basidiomycete fungus responsible for cryptococcosis, particularly in immunocompromised individuals. With increasing incidence rates globally, there is an urgent need for improved therapeutic strategies. This study presents the development of a fungal-targeted nanodrug delivery system utilizing a calcofluor white-phosphatidylethanolamine conjugate (CFW-PEc) to enhance the delivery and efficacy of amphotericin B against *C. neoformans*. We successfully formulated AmB-loaded CFW-PEc-ethosomes, which demonstrated favorable physicochemical properties, including suitable particle size, zeta potential, and high drug entrapment efficiency. *In vitro* antifungal assessments revealed that CFW-PEc-AmB-ethosomes exhibited superior antifungal activity compared to conventional AmB formulations, maintaining high inhibition rates at lower concentrations, while also demonstrating a favorable safety profile with reduced cytotoxicity. Additionally, *in vivo* studies in a mouse model of cryptococcal pneumonia illustrated remarkable reductions in fungal burdens and improved histopathological outcomes, attributing these effects to effective targeting of *C. neoformans* via CFW-PEc. Our findings underscore the potential of CFW-PEc in enhancing the therapeutic efficacy and safety profile of antifungal treatments, paving the way for advanced treatment strategies against cryptococcal pneumonia.

**IMPORTANCE:**

Cryptococcal pneumonia presents a significant global health burden with limited therapeutic options due to inherent toxicity and suboptimal bioavailability of conventional antifungal agents. This investigation demonstrates the innovative application of calcofluor white-phosphatidylethanolamine conjugate (CFW-PEc) to enhance amphotericin B (AmB) delivery via ethosomes for cryptococcal infection treatment. Our findings elucidate that CFW-PEc significantly potentiates the antifungal efficacy of AmB-loaded ethosomes against *Cryptococcus neoformans* while concomitantly mitigating associated cytotoxicity at optimal concentrations. In murine models of pulmonary cryptococcosis, this novel formulation achieved a remarkable 10-fold reduction in fungal burden compared to controls, while preserving pulmonary histoarchitecture and attenuating inflammatory responses. This delivery system’s integrated strategy of increasing antifungal effectiveness while reducing adverse effects marks a significant leap forward in developing safer and more targeted nanomaterial-mediated antifungal treatments. These results have profound implications for developing more efficacious and less toxic treatment modalities for cryptococcal pneumonia and potentially other invasive fungal infections.

## INTRODUCTION

*Cryptococcus neoformans* is a significant pathogen in the realm of mycology, recognized as a lethal encapsulated basidiomycete fungus responsible for severe pulmonary infections and life-threatening cryptococcosis (1, 2). Since 2009, the incidence of cryptococcosis has nearly doubled in Europe, North America, and Latin America, whereas other regions have experienced either a decrease or a stable number of cases (3). The risk of *C. neoformans* infections is particularly heightened in immunocompromised individuals, with an alarming 19% of all HIV-related deaths being attributed to this pathogenic fungus (4, 5). *C. neoformans* stands as a substantial global threat to human health, necessitating heightened attention and response strategies to mitigate its impact (3).

Cryptococcal infections typically initiate in the lungs following inhalation of environmental spores, where *C. neoformans* adapts rapidly to the pulmonary environment by producing a protective capsule and evading immune responses (2, 3). While amphotericin B is commonly used for severe manifestations like cryptococcal meningitis or disseminated disease, particularly in immunocompromised patients (4, 6), pulmonary cryptococcosis represents the primary entry point and can progress to systemic involvement if not addressed early. Our study focuses on lung involvement to explore targeted delivery strategies that could prevent dissemination from the initial infection site, potentially improving outcomes in high-risk populations such as those with HIV/AIDS (4). Infections typically initiate in lung tissues following the inhalation of environmental spores, where *C. neoformans* demonstrates remarkable adaptability. The fungus quickly modifies its morphology upon encountering pulmonary environments, producing a protective capsular structure and forming enlarged fungal cells known as Titan cells, alongside the elaboration of various virulence factors (7, 8). However, the management of cryptococcal infections is fraught with challenges, including the limited effectiveness of existing antifungal therapies, their associated toxicities, the versatile adaptation of *C. neoformans* in the host, and the emergence of resistance (9).

Novel clinical antifungal agents are urgently needed to overcome these challenges. In response, extensive research has focused on the development of innovative nanoparticle-mediated drug delivery systems. Among these, liposomal formulations of AmB have gained interest for encapsulating the drug, improving its pharmacological profile, reducing systemic toxicity, and decreasing the frequency of administration (10–12). Nonetheless, liposomal formulations of AmB face common drawbacks, including dose-limiting toxicities, particularly infusion-related reactions and renal toxicity, which may necessitate careful monitoring and dose adjustments. Additionally, these formulations may not achieve optimal concentrations at infection sites due to their distribution characteristics, potentially compromising their overall therapeutic effectiveness (13). Therefore, there is an urgent need for specialized nanoparticle-based delivery systems with targeting mechanisms aimed at *C. neoformans*. Such systems could utilize receptor-mediated endocytosis or unique microbial characteristics to ensure effective targeting and treatment of cryptococcosis, ultimately improving patient outcomes while minimizing harm to healthy tissues.

Fungal cell walls are primarily composed of a complex matrix of polysaccharides, proteins, and other components, which form a hierarchical structure that provides mechanical support and mediates interactions with the environment, including immune recognition by host organisms. The inner layer typically consists of covalently linked branched β-(1,3) glucans and chitin, which assemble into a fibrous network that helps withstand internal hydrostatic pressure (14). The outer layers of the cell wall can vary considerably among fungal species and may include highly mannosylated glycoproteins and other polysaccharides that serve to enhance structural integrity and influence immune responses (15, 16). *C. neoformans* exhibits distinct characteristics in its chitin composition compared to other fungi. While chitin serves as a structural backbone in the cell walls of many fungi, including yeasts like *Saccharomyces cerevisiae* and *Candida albicans*, the chitin in *C. neoformans* predominantly exists in a deacetylated form known as chitosan, which enhances its protective qualities (17). Importantly, *C. neoformans* contains a significantly high proportion of chitin in its cell wall, which is crucial for its physiological functions and immune evasion mechanisms (18). This is in contrast to various yeast species, such as *C. albicans*, which may exhibit multiple forms of chitin with relatively lower degrees of deacetylation (19). Furthermore, the chitin content in *C. neoformans* is integrated with a substantial capsule made up of glucuronoxylomannan and galactoxylomannan (20, 21), setting it apart from other fungal cell walls where chitin may not play a central role in the outer structure. This high concentration of chitin reflects the unique physiological and pathogenic adaptations of *C. neoformans*, emphasizing the critical role of cell wall composition in fungal biology and immune evasion strategies (22).

Calcofluor white (CFW) is a blue fluorescent dye commonly used in microbiological laboratories for the identification of fungi. It binds to chitin in the fungal cell wall and exhibits bright fluorescence under UV light (23–25). There is a competitive interaction with hydrogen bond sites between CFW and natural chitin, leading to the disruption of chitin assembly. Consequently, the integrity of the fungal cell wall is compromised, and fungal growth is inhibited (26–28). The CFW-phosphatidylethanolamine conjugate (CFW-PEc) synthesized in the earlier study possesses the capability to target chitin within fungal cell walls (29). Since this component is present in the cell wall of *C. neoformans*, it provides potential opportunities for the development of targeted nano-delivery systems for the treatment of cryptococcal pneumonia.

**Fig 1:**
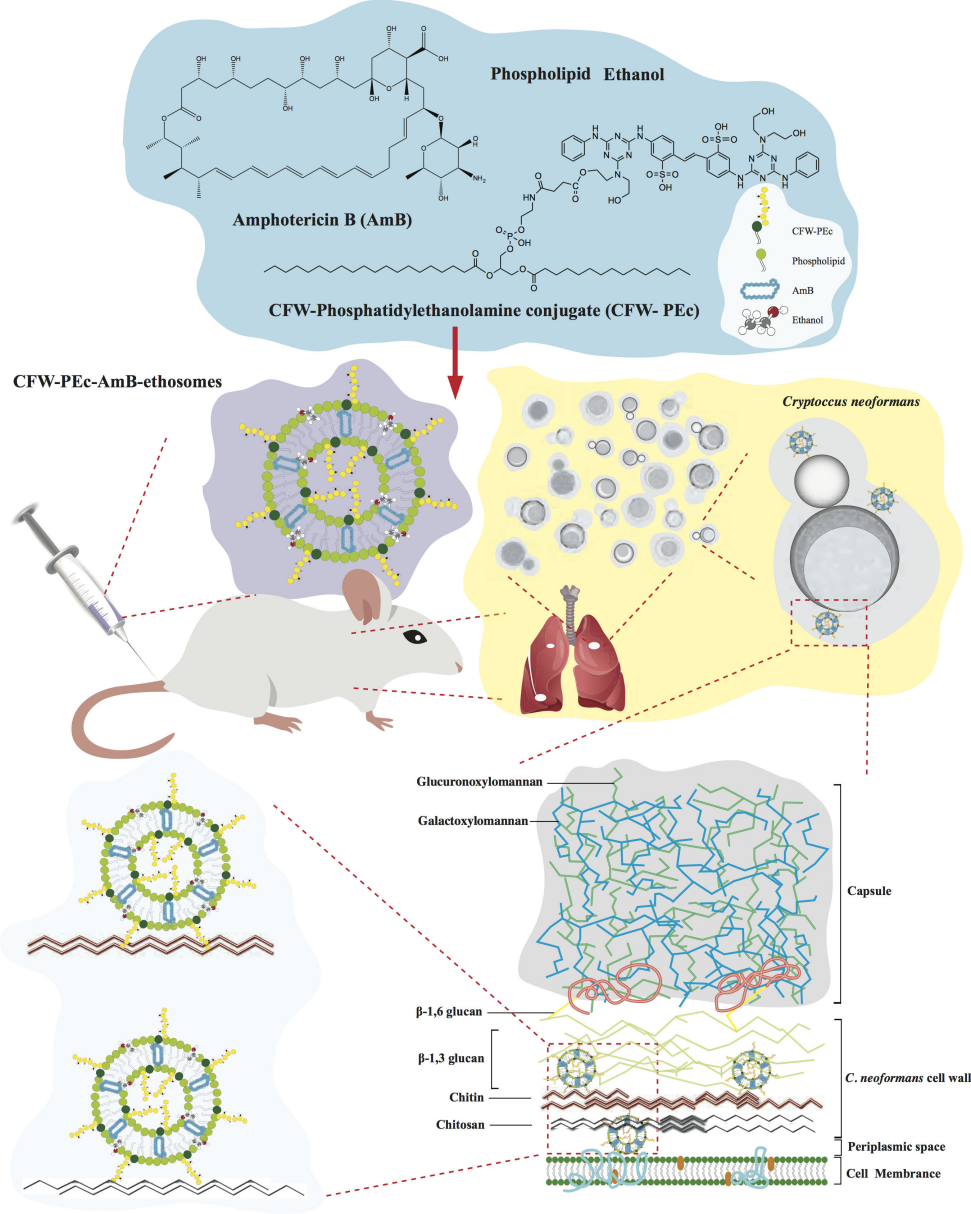
Therapeutic application of CFW-PEc-AmB-ethosomes in mice with cryptococcal pneumonia. CFW-PEc, a chitin-targeting compound, is formulated into ethosomes encapsulating AmB for intravenous administration to mice infected with *C. neoformans*. This pathogenic fungus poses significant risks, especially to immunocompromised individuals, due to its ability to form a robust biofilm at infection sites, consisting of polysaccharides like chitin and glucans that aid in immune evasion. CFW-PEc enhances the targeted delivery of AmB-encapsulated ethosomes to the infection sites via the bloodstream, improving drug localization and therapeutic efficacy. This strategy addresses the challenges of resistance and toxicity associated with conventional antifungal treatments, offering a promising approach for better outcomes in cryptococcal pneumonia management.

Recent advancements in targeted lipid-based delivery systems, such as liposomes analogous to ethosomes, have shown substantial promise in antifungal drug delivery, particularly for reducing toxicity and enhancing specificity in pulmonary fungal infections. For instance, Dectin-1 and Dectin-2 targeted amphotericin B liposomes exhibit stronger binding to fungal cell walls, including those of *C. neoformans*, leading to improved efficacy in pulmonary aspergillosis models and reduced fungal burden (30, 31). Similarly, DC-SIGN-targeted liposomes enhance binding to polysaccharide matrices of *C. neoformans*, resulting in superior antifungal activity and lower resistance at reduced doses (32). LysM-modified liposomes further increase targeting to fungal hyphae, demonstrating enhanced activity against *C. neoformans* with prolonged circulation via PEGylation (33). These developments align with our work in leveraging lipid carriers to overcome barriers like poor bioavailability and systemic toxicity in antifungal therapy. However, our CFW-PEc-AmB-ethosomes differ by incorporating a chitin-binding domain (CFW-PEc) for specific targeting of the *C. neoformans* cell wall, combined with ethanol-enhanced deformability for better pulmonary penetration, unlike the primarily receptor-mediated or PEG-dependent mechanisms in targeted liposomes. This distinction addresses a gap in ethosome-like applications, enabling more precise delivery in deep-seated lung infections while minimizing off-target effects. In this study (Fig. 1), we developed fungal-targeted nanodrug delivery systems utilizing CFW-PEc, which comprise AmB-loaded CFW-PEc-ethosomes (CFW-PEc-AmB-ethosomes). We thoroughly determined their physicochemical properties, including particle size, zeta potential, drug loading (DL) capacity, and entrapment efficiency. The *in vitro* antifungal activity of CFW-PEc-AmB-ethosomes against *C. neoformans* cells was evaluated. We specifically characterized the drug delivery effectiveness of CFW-PEc-ethosomes in *C. neoformans* cells *in vitro* to elucidate the relationship between the amount of CFW-PEc and the delivery efficiency of these ethosomes. Furthermore, we investigated the antifungal efficacy of CFW-PEc-AmB-ethosomes in a mouse model of cryptococcal pneumonia, alongside an assessment of their biosafety *in vitro*. Our findings demonstrate that the biomaterial CFW-PEc effectively targets *C. neoformans* by binding to chitin in the cell walls, significantly enhancing the delivery efficiency and antifungal potency of nanoparticle-based AmB therapies. This advancement represents a promising strategy for potential clinical application in the treatment of cryptococcal pneumonia.

## MATERIALS AND METHODS

### Materials and strains

The study utilized several key materials, including AmB, Coumarin 6 (C6), phospholipids, anhydrous ethanol, dimethyl sulfoxide (DMSO), phosphate-buffered saline (PBS), and CCK-8, all sourced from Sigma-Aldrich (St. Louis, MO, USA). Purified water was obtained using a Merck Millipore Ultrapure Water System (Darmstadt, Germany) for all experimental procedures. The remaining chemicals and reagents were of analytical grade or higher. CFW-PEc was sourced from prior research efforts (29). The strains employed in this study were the sequenced reference strain *C. neoformans H99*, which was acquired from the American Type Culture Collection. The fungal cells were cultured on Yeast-Peptone-Dextrose (YPD) plates, which contained 1% yeast extract, 2% peptone, 2% dextrose, and 2% agar, as well as in YPD broth composed of 1% yeast extract, 2% peptone, and 2% dextrose.

### Preparation of C6 and AmB-loaded ethosomes

AmB-loaded ethosomes (AmB-ethosomes) and CFW-PEc-AmB-ethosomes were prepared using the ethanol injection method. Initially, 0.5 mg of AmB was dissolved in 150 µL of DMSO under ultrasonic conditions. Subsequently, 67 mg of phospholipids were dissolved in 1 mL of anhydrous ethanol, also using ultrasound. In this ultrasonic environment, the previously prepared DMSO solution (150 µL) was mixed with the anhydrous ethanol solution (150 µL) and then injected into 700 µL of deionized water to obtain 1 mL of AmB-ethosomes. For the preparation of CFW-PEc-AmB-ethosomes, 10 mg of CFW-PEc was dissolved in 1 mL of DMSO. Similar to the previous procedure, 0.5 mg of AmB was dissolved in DMSO, and 67 mg of phospholipids was dissolved in 1 mL of anhydrous ethanol. The DMSO solution was mixed with the anhydrous ethanol, and the previously prepared CFW-PEc solution was added and thoroughly mixed. The resulting mixture was then injected into deionized water to yield 1 mL of CFW-PEc (1 µM)-AmB-ethosomes. This process was similarly applied to prepare CFW-PEc (0.1 µM)-AmB-ethosomes and CFW-PEc (0.01 µM)-AmB-ethosomes. C6 was dissolved in anhydrous ethanol at a concentration of 100 µg/mL and stored in the dark at 4°C. Seven to eight milligram of phospholipids were then dissolved in 200 µL of the reserved C6 solution under ultrasonic conditions. This solution was slowly injected into 800 µL of deionized water to obtain C6-ethosomes. The same method was used to prepare CFW-PEc (0.01 µM)-C6-ethosomes, CFW-PEc (0.1 µM)-C6-ethosomes, and CFW-PEc (1 µM)-C6-ethosomes.

### Characterization of physicochemical properties and stability of AmB-ethosomes and CFW-PEc-AmB-ethosomes

The particle size distribution and surface zeta potential of CFW-PEc (0.01 µM)-AmB-ethosomes, CFW-PEc (0.1 µM)-AmB-ethosomes, CFW-PEc (1 µM)-AmB-ethosomes, and AmB-ethosomes were measured using a Zetasizer Nano ZS. The prepared samples were diluted 10-fold with deionized water and filtered through a 0.22 µm membrane before measurement. The particle size distribution and zeta potential were measured on days 1, 3, 5, 7, 9, 11, and 14 to assess stability.

### Transmission electron microscopy observation

The morphology of CFW-PEc (0.01 µM)-AmB-ethosomes, CFW-PEc (0.1 µM)-AmB-ethosomes, CFW-PEc (1 µM)-AmB-ethosomes, and AmB-ethosomes was examined using transmission electron microscopy (TEM). A 10 µL sample was placed on a copper grid coated with a sulfur membrane and fixed for 8–10 minutes. The excess sample was removed using filter paper, and the sample was stained with 10 µL of 1% phosphotungstic acid solution for 2 minutes. After drying at room temperature, the sample was observed under the TEM for morphological characteristics.

### Determination of encapsulation efficiency and drug loading of CFW-PEc-AmB-ethosomes and AmB-ethosomes

A standard curve for AmB was established using a C18 reverse phase chromatographic column (4.6 × 250 mm, 5 µm) with an acetonitrile: disodium EDTA mobile phase in a ratio of 38:62, a flow rate of 1.0 mL/min, a detection wavelength of 405 nm, and a column temperature of 30°C. AmB was diluted in acetonitrile, and the peak area was measured to construct the standard curve. To determine the encapsulation efficiency (EE) and DL of AmB, 1 mL of CFW-PEc-AmB-ethosomes and AmB-ethosomes was centrifuged at 10,000 rpm for 1 hour using an ultrafiltration tube (10 kDa). The filtrate was collected, and the concentration of free drug was measured using high-performance liquid chromatography (HPLC). A volume of 100 µL of ethosomes was mixed with 900 µL of methanol and subjected to ultrasound for 15 minutes; the total drug content was measured using HPLC. The EE and DL were calculated according to a previous description (29, 34).

### Antifungal activity of CFW-PEc-AmB-ethosomes and AmB-ethosomes against *C. neoformans*

The antifungal efficacy of the formulations against *C. neoformans* was evaluated in YPD broth as per the protocols provided by the National Committee for Clinical Laboratory Standards (M27-A3). Serial dilutions of CFW-PEc ranged from 0.03 μM to 256 µM, while CFW-PEc (0.01–1 µM)-AmB-ethosomes and AmB-ethosomes (0.015–32 µg/mL) were prepared in YPD broth. A single colony of *C. neoformans H99* was inoculated in 5 mL of YPD broth and incubated overnight at 30°C with shaking at 200 rpm. Following overnight incubation, 100 µL of the yeast culture was diluted 10-fold in YPD broth to measure the optical density (OD) at 600 nm using a UV spectrophotometer, and the cell suspension was diluted to 1 × 10^5^ CFU/mL for subsequent assays. A 96-well plate was prepared with 100 µL of the prepared samples in YPD broth. Subsequently, 100 µL of the diluted yeast suspension (1 × 10^5^ CFU/mL) was added to the wells. Control wells received 200 µL of YPD broth, while antagonist control wells received 100 µL of yeast suspension and 100 µL of YPD broth. The plates were incubated at 30°C with shaking at 200 rpm for 24 hours. The absorbance was measured at 600 nm using a plate reader to calculate the inhibition rate of the samples against *C. neoformans*.

The growth curve assay was conducted to further evaluate the antifungal activity of the formulations. Using the same culture method, *C. neoformans H99* was grown overnight and diluted to 4 × 10^5^ CFU/mL in fresh YPD broth. The diluted culture (50 mL) was distributed into sterile Erlenmeyer flasks, followed by the addition of test formulations: AmB-ethosomes, CFW-PEc (0.01 µM)-AmB-ethosomes, CFW-PEc (0.1 µM)-AmB-ethosomes, and CFW-PEc (1 µM)-AmB-ethosomes, with a final AmB concentration of 0.5 µg/mL in each treatment group. An equivalent volume of YPD broth was added to the control group. All flasks were incubated at 30°C with shaking at 200 rpm, with three replicates per group. Growth curves were generated by measuring OD at specific time points (2, 4, 6, 8, 10, 12, and 24 hours post-inoculation). At each time point, 100 µL of culture was sampled from each flask (after gentle mixing to ensure homogeneous cell distribution) and diluted with 900 µL of YPD broth. The OD values were measured using a UV spectrophotometer at 600 nm.

### *In vitro* cytotoxicity of CFW-PEc and CFW-PEc-AmB-ethosomes

All experiments involving human cells were approved by the Research Ethics Committees of Binzhou Medical University (Approval No. 2024-118). The L929 cell lines were authenticated via an short tandem repeat (STR) profile conducted by Shanghai Biowing Applied Biotechnology Co., Ltd. A sterile solution of 0.1 mM CFW and CFW-PEc was prepared and filtered through a 0.22 µm membrane. The stock solution was diluted 10-fold with Dulbecco's Modified Eagle's Medium (DMEM) to achieve a concentration of 10 µM, and further dilutions were made to obtain working concentrations of 5 µM, 1 µM, 0.5 µM, and 0.1 µM. L929 cells were diluted to 5 × 10^4^ CFU/mL, and 180 µL of the cell suspension was added to each well of a 96-well plate alongside controls containing cell suspension and blank DMEM medium (180 µL each). The plates were incubated overnight at 37°C in a 5% CO_2_ atmosphere. After overnight incubation, 20 µL of the prepared samples was added to the respective wells. The control wells received 20 µL of DMEM. The plates were incubated for an additional 24 hours, after which 10 µL of CCK-8 was added to each well and incubated for 3–4 hours. The absorbance at 450 nm was measured to determine cell viability. The same procedure was followed to assess cell viability in response to AmB, AmB-ethosomes, and CFW-PEc (0.01–1 µM)-AmB (0.5 µg/mL)-ethosomes.

### Delivery efficiency of CFW-PEc-C6-ethosomes in *C. neoformans* cells

A single colony of *C. neoformans H99* was inoculated in 5 mL of YPD medium and incubated overnight at 30°C with shaking at 200 rpm. Following overnight incubation, *C. neoformans H99* was harvested and washed twice with PBS (3,000 rpm for 5 minutes). C6-loaded ethosomes were diluted to a concentration of 0.3 µg/mL. *C. neoformans* cells were diluted to 1 × 10^5^ CFU/mL and added to a 24-well plate (1.8 mL per well). C6-ethosomes and CFW-PEc (0.01–1 µM)-C6-ethosomes were added to both control and experimental groups at various time points (3 h, 6 h, and 9 h) to allow for co-incubation. Following this incubation, the mixture was centrifuged at 3,000 rpm for 5 minutes to collect 400 µL of the *C. neoformans* cells, which were washed twice with PBS and then resuspended in 10 µL of PBS for laser confocal microscopy (Leica TCS SPE) imaging. Fluorescence images were obtained using two different UV excitation wavelengths, capturing emissions at 490 ± 10 nm for C6 and 435 ± 10 nm for CFW-PEc. Image J software was used to calculate the average fluorescence intensity by dividing the total fluorescence signal by the area of the cells.

### *In vivo* antifungal efficacy of CFW-PEc-AmB-ethosomes against cryptococcal pneumonia

Animal experiments adhered to the guidelines set forth in NIH Publications No. 8023, revised in 1978, and received approval from the Research Ethics Committees of Binzhou Medical University (Approval No. 2024-209). Ethical review for laboratory animal welfare followed the Chinese National Guidelines (GB/T 35892-2018). *C. neoformans H99* cells were prepared as previously described. After a 100-fold dilution, 10 µL was plated on a counting chamber, and the number of colonies was observed under a microscope. The yeast suspension was adjusted to 2 × 10^6^ CFU/mL. Four- to five-week-old Kunming mice weighing 14–16 g were obtained from Jinan Pengyue Experimental Animal Breeding Co., Ltd. A total of 54 Kunming mice were randomly divided into six groups, each containing nine mice. The mice were anesthetized via intraperitoneal injection of 3% hydrous chloral hydrate. Following anesthesia, 50 µL of the *C. neoformans* cell suspension was administered intranasally in order to establish a model of cryptococcal pneumonia.

Over a period of 7 days post-infection, treatment with CFW-PEc (0.01–1 µM)-AmB-ethosomes and AmB-ethosomes was administered, with a dose of 0.8 mg/kg AmB per average body weight of mice. After 7 days of treatment, the mice were euthanized by cervical dislocation, and the lungs were excised, homogenized in 10 mL of PBS, and diluted 100-fold in PBS. A 100 µL aliquot of the diluted solution was plated on YPD solid medium containing 100 µg/mL of ampicillin and 50 µg/mL of kanamycin and incubated at 30°C for 2 days for colony counting.

For histopathological evaluation, three mice from each group were anesthetized, and their thoracic cavities were carefully opened using sterile scissors to expose the heart for cardiac perfusion. A needle was inserted into the left ventricle, and a small incision was made in the right atrium for the infusion of physiological saline, allowing the blood to be cleared until the lungs appeared white. Subsequently, the lungs were fixed in 4% paraformaldehyde, and hematoxylin and eosin (H&E) staining was performed for histological assessment.

### Statistical analysis

All experiments were performed independently a minimum of three times to ensure reproducibility, and the results are presented as mean ± SD. Statistical analyses were conducted using analysis of variance (ANOVA) followed by Tukey’s post hoc test (SPSS 19.0; IBM, Armonk, NY, USA). A *P*-value of ≤0.05 was considered statistically significant.

## RESULTS

### Characterization of CFW-PEc-AmB-ethosomes

**Fig 2:**
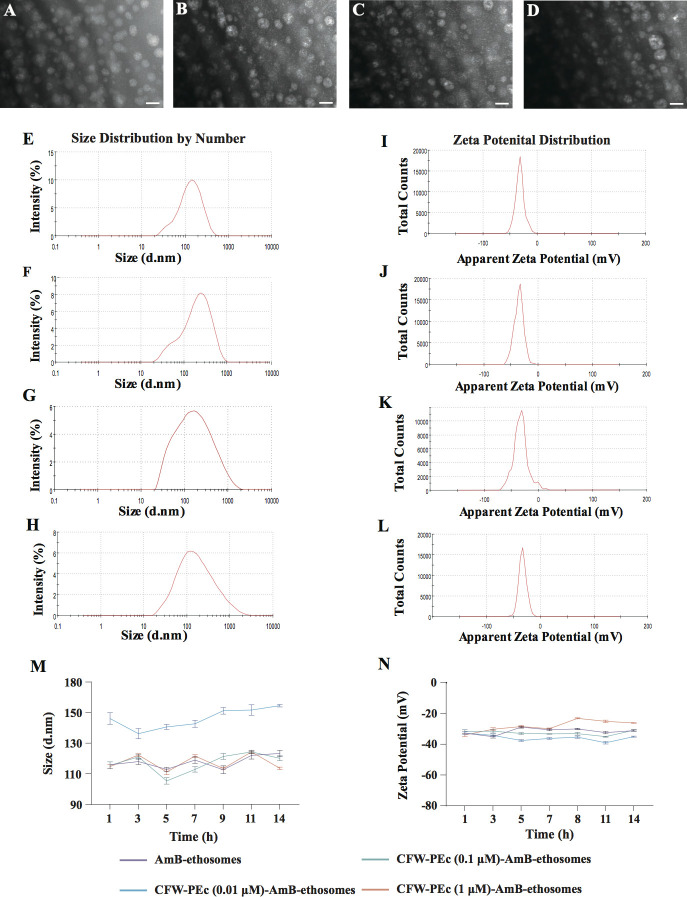
Morphological characterization of AmB-ethosomes and CFW-PEc (0.01 μM–1 µM)-AmB-ethosomes. TEM images reveal that AmB-ethosomes (**A**), CFW-PEc (0.01 µM)-AmB-ethosomes (**B**), CFW-PEc (0.1 µM)-AmB-ethosomes (**C**), and CFW-PEc (1 µM)-AmB-ethosomes (**D**) exhibit a nearly spherical shape and uniform distribution, with no observable aggregation, indicating the successful preparation of the formulations. All scale bars are 200 nm. Particle size and zeta potential analysis of AmB-ethosomes and CFW-PEc (0.01–1 µM)-AmB-ethosomes. (**E**) Size distribution of AmB-ethosomes and (**F–H**) CFW-PEc-AmB-ethosomes at concentrations of 0.01 µM, 0.1 µM, and 1 µM, respectively. Average particle sizes are presented in Table 1. (**I–L**) Zeta potential distributions for AmB-ethosomes (**I**) and CFW-PEc-AmB-ethosomes at varying concentrations (**J–L**). Values indicate all formulations possess negative surface charges, suggesting good stability. (**M and N**) Particle size and zeta potential of CFW-PEc (0.01–1 µM)-AmB-ethosomes following a 2-week storage period at 25°C. Vertical bars indicate mean ± SD from three experiments.

The CFW-PEc (0.01–1 µM)-AmB-ethosomes were successfully prepared as illustrated in Fig. 1. The particle morphologies of both AmB-ethosomes and CFW-PEc (0.01–1 µM)-AmB-ethosomes were examined using transmission electron microscopy, as shown in Fig. 2A through D. The AmB-ethosomes alone exhibited comparable morphology, preserving a spherical shape and demonstrating excellent dispersibility without any signs of aggregation (Fig. 2A). Likewise, the CFW-PEc (0.01–1 µM)-AmB-ethosomes exhibited a nearly spherical shape, uniform dispersion, and no signs of particle aggregation (Fig. 2B through D). These results suggest that the addition of CFW-PEc material had no negative impact on the morphology of the AmB-loaded ethosomes.

The particle size and zeta potential of nanoparticles were characterized using dynamic light scattering. The size distributions for AmB-ethosomes and CFW-PEc (0.01–1 µM)-AmB-ethosomes are illustrated in Fig. 2E through H, with Fig. 2E showing AmB-ethosomes and Fig. 2F through H representing CFW-PEc at concentrations of 0.01 µM, 0.1 µM, and 1 µM, respectively. Average particle sizes are summarized in Table 1. The measured particle sizes for AmB-ethosomes and CFW-PEc (0.01–1 µM)-AmB-ethosomes were 165.63 ± 0.71 nm, 182.10 ± 0.37 nm, 187.50 ± 0.22 nm, and 195.60 ± 0.89 nm, respectively. These results indicate a gradual increase in particle size with higher CFW-PEc concentrations, suggesting that the addition of CFW-PEc contributes to larger particle sizes, potentially affecting the delivery and release characteristics of the antifungal agent. The zeta potential values for these formulations are presented in Fig. 2I through L, with Fig. 2I displaying the distribution for AmB-ethosomes and Fig. 2J through L for CFW-PEc at the same concentrations. Average zeta potential values are also listed in Table 1. The zeta potential values for CFW-PEc (0.01–1 µM)-VRC-ethosomes and VRC-ethosomes were −32.80 ± 0.94 mV, −32.77 ± 0.74 mV, −31.63 ± 0.93 mV, and −33.97 ± 0.79 mV, indicating that all formulations exhibit a negative surface charge. These negative zeta potential values are significant indicators of formulation stability, as they represent the degree of electrostatic repulsion between similarly charged particles in the dispersion. Higher negative values (generally below −30 mV) indicate strong repulsive forces between particles, which effectively prevent aggregation and ensure long-term stability of the nano formulation (35, 36).

**TABLE 1:** Therapeutic properties of nanoparticles (*n* = 3)^*a*^^,^^*b*^

	AmB-ethosomes	CFW-PEc (0.01 µM)-AmB-ethosomes	CFW-PEc (0.1 µM)-AmB-ethosomes	CFW-PEc (1 µM)-AmB-ethosomes
Size (nm)	165.63 ± 0.71	182.10 ± 0.37	187.50 ± 0.22	195.60 ± 0.89
Zeta Potential (mV)	−32.80 ± 0.94	−32.77 ± 0.74	−31.63 ± 0.93	−33.97 ± 0.79
EE (%)	84.12 ± 0.16	81.74 ± 0.29	80.99 ± 1.12	79.29 ± 0.43
DL capacity (%)	10.34 ± 0.05	15.84 ± 0.06	11.98 ± 0.17	16.86 ± 0.04

^*a*^ EE, entrapment efficiency; DL, drug loading capacity.

^*b*^ All data expressed as means ± standard deviations.

EE and DL are critical parameters for assessing drug delivery systems (37). As shown in Table 1, the AmB-ethosomes exhibited an EE of 84.12% ± 0.16% and a DL of 10.34% ± 0.05%. In contrast, the EE of CFW-PEc (0.01–1 µM)-AmB-ethosomes varied between 79.29% ± 0.43% and 81.74% ± 0.29%, while the DL ranged from 11.98% ± 0.17% to 16.86% ± 0.04%. Moreover, Fig. 2M and N demonstrate that there were no significant changes in the particle size and zeta potential of CFW-PEc (0.01–1 µM)-AmB-ethosomes after a 2-week storage period at 25°C. This observation indicates that the formulations possess excellent storage stability.

### *In vitro* assessment of antifungal activity of CFW-PEc and CFW-PEc-AmB-Ethosomes

**Fig 3:**
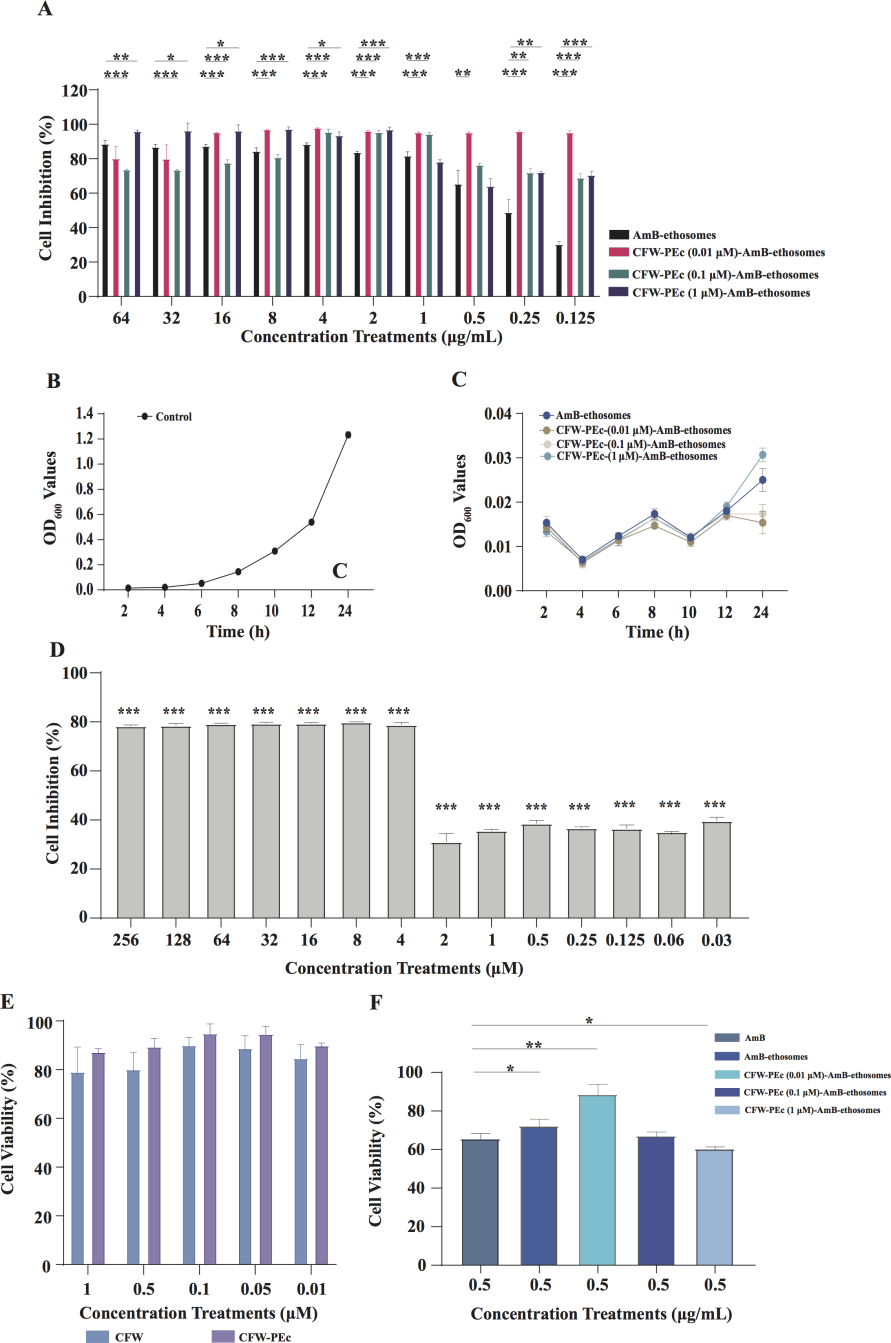
Antifungal activity of AmB-ethosomes and CFW-PEc (0.01–1 µM)-AmB-ethosomes against *C. neoformans in vitro*. (**A**) Inhibition rates of CFW-PEc (0.01–1 µM)-AmB-ethosomes and AmB-ethosomes at various concentrations, showing superior antifungal activity of CFW-PEc formulations, particularly at lower concentrations. (**B and C**) Growth curve assays showing OD values over a 24-hour incubation period for control and treatment groups. (**D**) Consistent inhibition rates of CFW-PEc at concentrations from 256 μM to 0.03 µM, indicating effective antifungal effects across the tested range. (**E**) Viability of L929 cells treated with CFW and CFW-PEc, demonstrating the favorable safety profile of CFW. The CFW-PEc retains the safety characteristics of CFW in L929 cells, indicating its potential for safe application in therapeutic settings. (**F**) Cytotoxicity assessment of CFW-PEc (0.01–1 µM)-AmB (0.5 µg/mL) on L929 cells at 0.5 µg/mL. Vertical bars indicate mean ± SD from three experiments, with significant differences highlighted (*P ≤ 0.05; **P ≤ 0.01; ***P ≤ 0.001).

The antifungal activity of AmB ethosomal formulations was evaluated through an *in vitro* antifungal test conducted in YPD broth containing *C. neoformans* cells at various concentrations. The inhibition rates of AmB-ethosomes and CFW-PEc (0.01–1 µM)-AmB-ethosomes against *C. neoformans* cells are summarized in Fig. 3A. AmB-ethosomes exhibited a concentration-dependent inhibition, with rates ranging from 88.74% at 64 µg/mL to 30.44% at 0.125 µg/mL. In contrast, CFW-PEc (0.01 µM)-AmB-ethosomes demonstrated consistently high inhibition rates, exceeding 95% from 16 μg/mL to 0.125 μg/mL, contrasting with the AmB-ethosomes alone, which showed reduced efficacy at similar concentrations. CFW-PEc (0.1 µM)-AmB-ethosomes exhibited moderate inhibition, with rates between 73.80% and 95.65%, while CFW-PEc (1 µM)-AmB-ethosomes achieved the highest inhibition rates, particularly at elevated concentrations, peaking at 97.34% at 8 µg/mL. Notably, CFW-PEc (0.01 µM)-AmB-ethosomes exhibited superior antifungal activity compared to other formulations, likely attributed to their effective drug delivery system. The enhanced activity at lower concentrations may be attributed to optimal molecular arrangement, leading to enhanced stability and drug delivery efficiency, whereas higher concentrations might cause molecular aggregation that interferes with delivery system performance. Overall, these results indicate that while higher concentrations enhance antifungal activity, CFW-PEc formulations effectively maintain substantial inhibition, especially at lower concentrations.

To further confirm the antifungal efficacy, a growth curve assay was performed over a 24-hour period, as illustrated in Fig. 3B and C. The control group exhibited a typical sigmoid growth curve of *C. neoformans*, with OD values progressively increasing from 0.015 at 2 hours to 1.233 at 24 hours post-inoculation. In contrast, all treatment groups demonstrated significant growth inhibition throughout the incubation period. The AmB-ethosomes group maintained low OD values ranging from 0.015 at 2 hours to 0.025 at 24 hours. Notably, the CFW-PEc (0.01 µM)-AmB-ethosomes group showed the most potent growth inhibition, with OD values consistently remaining below 0.018 throughout the 24-hour period, culminating in the lowest final OD (0.015) among all treatment groups. The CFW-PEc (0.1 µM)-AmB-ethosomes group displayed similar inhibitory effects with final OD values of 0.017, while the CFW-PEc (1 µM)-AmB-ethosomes group showed slightly higher OD values (0.031) at 24 hours. These growth curve results corroborate the inhibition rate findings, confirming that the combination of CFW-PEc with AmB-ethosomes, particularly at the 0.01 µM concentration, provides enhanced and sustained antifungal activity against *C. neoformans*.

To evaluate the antifungal activity of CFW-PEc against *C. neoformans* cells, an *in vitro* antifungal assay was also performed in YPD broth. The results, illustrated in Fig. 3D, show consistent inhibition rates across concentrations from 256 μM to 4 µM. Overall, the inhibition rates remained relatively stable, consistently ranging from approximately 78% to 80%. When extended to lower concentrations from 2 μM to 0.03 µM, a significant decrease in inhibition rates was observed, with values ranging from 31% to 39.6%. These results indicate effective antifungal activity of CFW-PEc against *C. neoformans* cells at higher concentrations (256–4 µM), with a marked reduction in efficacy at lower concentrations (2 µM and below).

### Cytotoxicity of CFW-PEc and CFW-PEc-AmB-ethosomes *in vitro*

An *in vitro* cytotoxicity study was performed to assess the safety profile of CFW-PEc and CFW-PEc-AmB-ethosomes in mammalian cells. The viability of L929 cells treated with CFW-PEc was compared to that of cells treated with CFW, as illustrated in Fig. 3E. At a concentration of 1 µM, the cell viability for L929 cells exposed to CFW-PEc was approximately 87.46%, while it increased to about 95.03% at a concentration of 0.1 µM. In contrast, the cell viability for CFW at the same concentrations was 79.29% at 1 µM and 90.21% at 0.1 µM. Although differences in cell viability between CFW and CFW-PEc treatment were observed, there was no statistical difference between the two groups. These findings indicate that CFW-PEc exhibits a positive safety profile *in vitro.*

The cell viability of L929 cells exposed to CFW-PEc (0.01–1 µM)-AmB (0.5 µg/mL)-ethosomes was compared to that of cells treated with a PBS solution, as illustrated in Fig. 3F. The results reveal that the inclusion of ethosomes significantly increases cell viability compared to AmB alone, with AmB (0.5 µg/mL)-ethosomes showing a viability of 72.52%. This indicates that ethosomes can reduce cytotoxicity, but the improvement is limited, as viability remains below that of the CFW-PEc formulations. Notably, CFW-PEc at a concentration of 0.01 µM combined with AmB (0.5 µg/mL)-ethosomes achieved the highest cell viability (88.84%). This suggests a protective effect of CFW-PEc on L929 cells, enhancing their survival even in the presence of AmB. Conversely, the formulation containing CFW-PEc at 1 µM showed decreased viability (60.66%), indicating that higher concentrations may induce cytotoxic effects. Similarly, CFW-PEc at 0.1 µM also resulted in moderate viability (67.33%), underscoring that while CFW-PEc can boost cell survival, excessive amounts can be detrimental. Overall, CFW-PEc appears to enhance the safety profile of AmB at lower concentrations, potentially minimizing cytotoxicity.

### Cellular uptake of CFW-PEc-C6-ethosomes in *C. neoformans* cells

**Fig 4:**
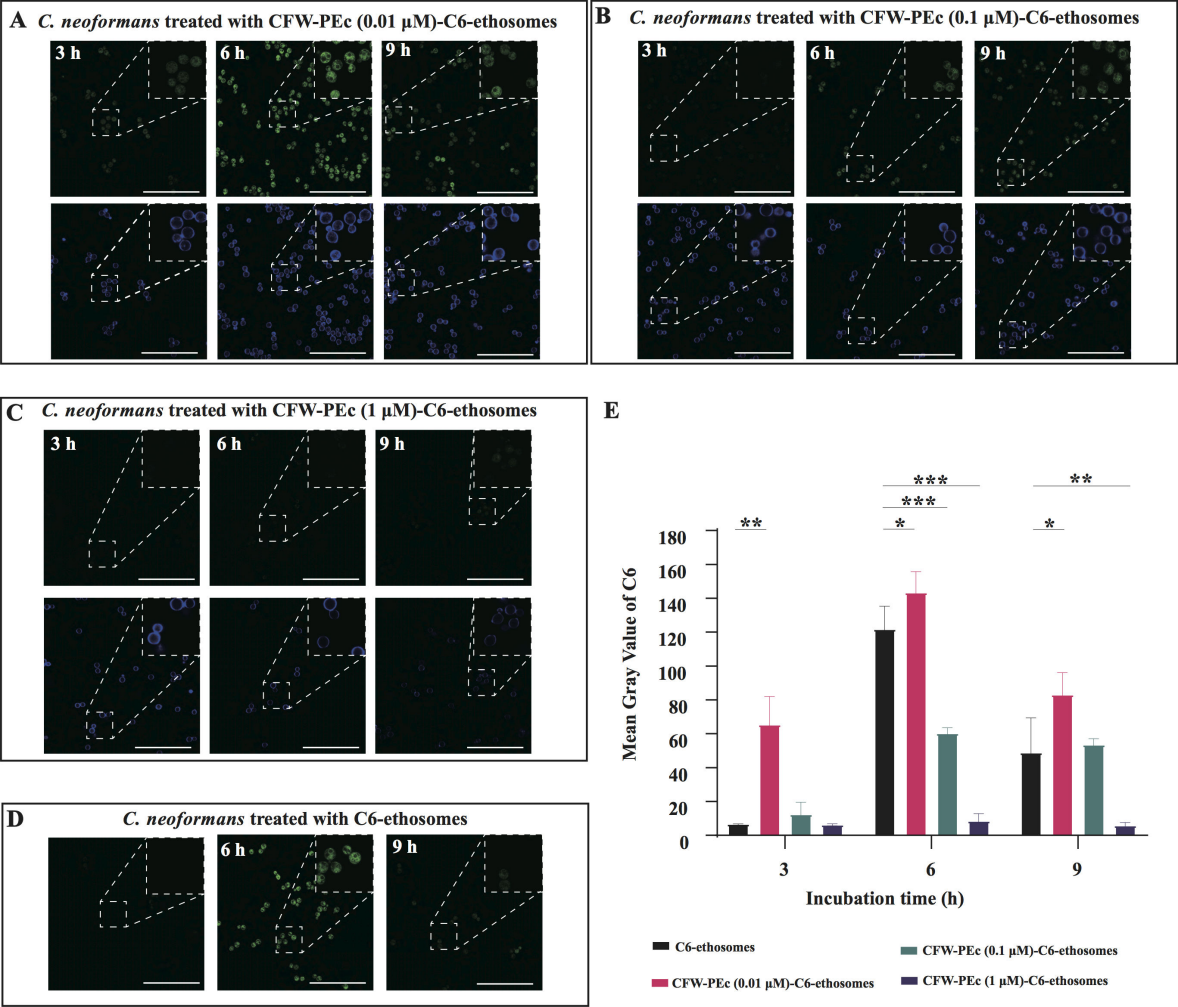
(**A–D**) Incubation of the *C. neoformans* cells with CFW-PEc (0.01 µM)-C6-ethosomes, CFW-PEc (0.1 µM)-C6-ethosomes, CFW-PEc (1 µM)-C6-ethosomes, or C6-ethosomes alone for durations of 3, 6, and 9 hours in YPD broth. Fluorescence microscopy images were captured using a Leica TCS SPE, displaying dual fluorescence channels: green from C6 and blue from CFW-PEc excitation. All images include scale bars of 50 µm. The white dashed lines indicate the magnified regions. All regions are magnified at the same scale. (**E**) Mean gray values of fluorescence intensity for different formulations over time, highlighting the concentration-dependent uptake characteristics and optimal internalization profiles for lower CFW-PEc concentrations. Vertical bars represent mean ± SD from three experiments at each time point, with significance levels indicated (*P ≤ 0.05; **P ≤ 0.01).

The cellular uptake of CFW-PEc-C6-ethosomes in *C. neoformans* cells was investigated using confocal microscopy. Figure 4A thorough D presents confocal micrographs that illustrate the uptake of C6-loaded ethosomes by *C. neoformans* cells. The results showed that in cells treated with CFW-PEc (0.01 µM)-C6-ethosomes (Fig. 4A), the fluorescence intensity of C6 peaked at 6 hours and then decreased by 9 hours. In contrast, the groups treated with CFW-PEc (0.1 µM)-C6-ethosomes (Fig. 3B) exhibited maximum fluorescence intensity at 6 hours, which remained stable until 9 hours. Meanwhile, the groups receiving CFW-PEc (1 µM)-C6-ethosomes (Fig. 4C) showed a lower and more consistent fluorescence intensity from 3 to 9 hours. Notably, the peak fluorescence intensity for all three formulations was reached at 6 hours in the CFW-PEc (0.01 µM)-C6-ethosome group. Additionally, typical blue fluorescence from CFW-PEc was observed outside the cell walls of *C. neoformans*. In the groups treated with C6-ethosomes (Fig. 4D), the fluorescence intensity pattern of C6 was comparable to that of the CFW-PEc (0.01 µM)-C6-ethosome group. The mean gray values of fluorescence intensity of C6, measured by ImageJ (Fig. 4E), revealed significant variations in the cellular uptake of dye-loaded nanoparticles over time. The CFW-PEc (0.01 µM)-C6-ethosomes exhibited the highest mean gray values at 6 hours, indicating robust cellular uptake, with a notable increase from 3 to 6 hours before declining at 9 hours. In contrast, the uptake of CFW-PEc (0.1 µM)-C6-ethosomes and CFW-PEc (1 µM)-C6-ethosomes was substantially lower overall. Meanwhile, C6-ethosomes also demonstrated substantial uptake, particularly at 6 hours, closely mirroring the performance of the lower amount CFW-PEc formulation.

### *In vivo* antifungal efficacy of CFW-PEc-AmB-ethosomes

**Fig 5:**
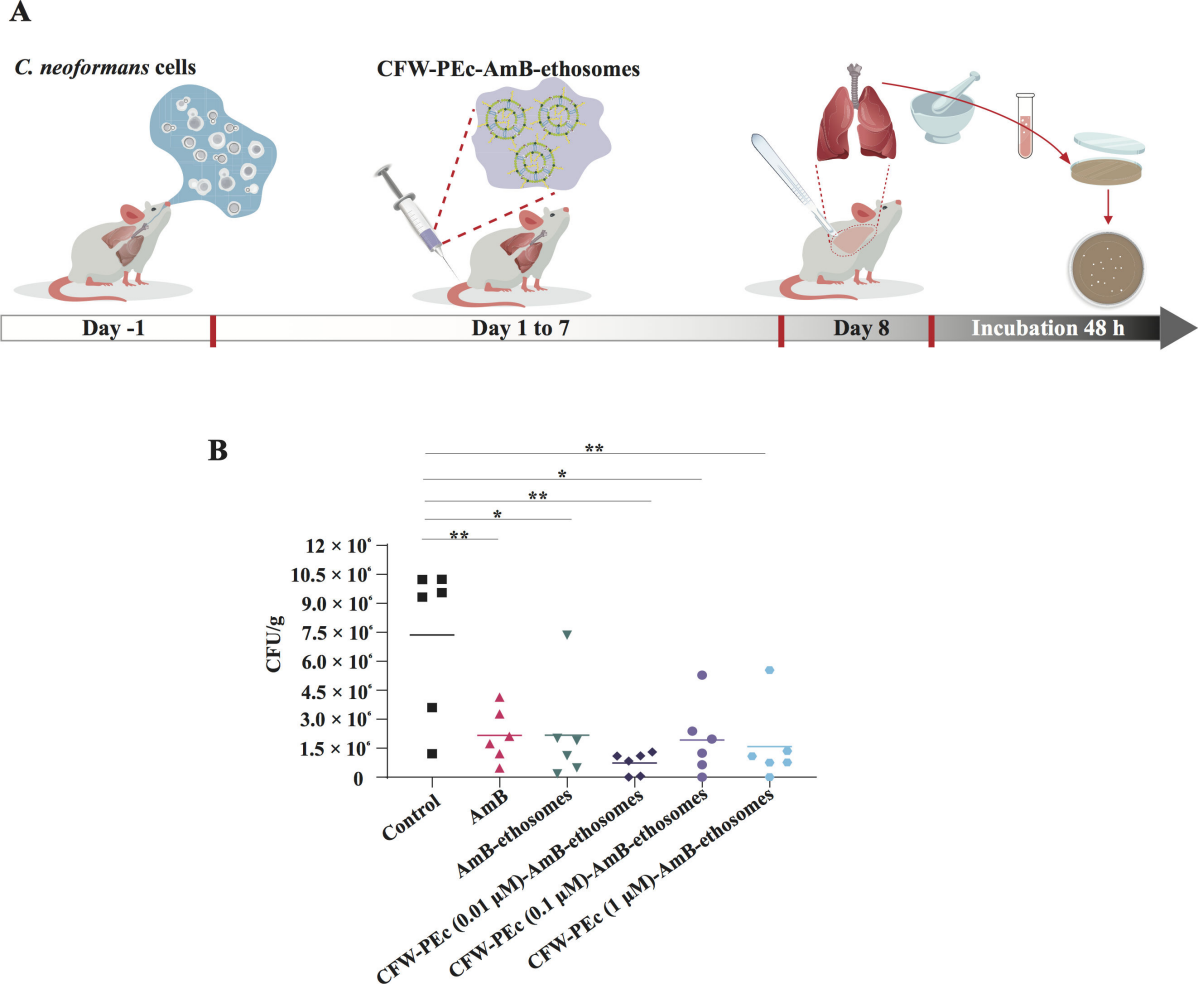
*In vivo* antifungal efficacy of CFW-PEc (0.01–1 µM)-AmB-ethosomes in mice infected with *C. neoformans*. (**A**) Diagram illustrating the experimental procedure involving intravenous administration of varying formulations. (**B**) Quantification of fungal burdens post-treatment, demonstrating significant reductions in CFU counts from lung tissues in mice treated with CFW-PEc (0.01–1 µM)-AmB-ethosomes, particularly noteworthy for the CFW-PEc (0.01 µM)-AmB-ethosomes group, indicating enhanced therapeutic efficacy (*P ≤ 0.05; **P ≤ 0.01).

To assess the effectiveness of CFW-PEc in enhancing the efficacy of AmB-loaded ethosomes against cryptococcal pneumonia, Kunming mice were infected with 1 × 10^5^ CFU of *C. neoformans H99* via intranasal administration. Five groups of Kunming mice were treated with 0.8 mg/kg of AmB in various formulations, while the control group received an appropriate PBS solution. Eight days post-infection, the lung tissues of the mice were homogenized to quantify CFU. The experimental procedure is illustrated in Fig. 5A, and the results are summarized in Fig. 5B. In comparison to the control group, significant reductions in fungal burdens were observed in the lungs of mice treated with AmB and AmB-loaded ethosomes. The CFU counts in the AmB-ethosomes group were approximately 3.4-fold lower than those in the control group, suggesting a moderate enhancement in efficacy. Notably, the groups treated with CFW-PEc (0.01–1 µM)-AmB-ethosomes exhibited the most pronounced reductions. Specifically, the CFW-PEc-(0.01 µM)-AmB-ethosomes group demonstrated a remarkable decrease in CFU counts to 0.73 × 10^6^ CFU/g, indicating an impressive 10-fold reduction compared to the control. When comparing the CFW-PEc-(0.01 µM)-AmB-ethosomes to the AmB-ethosomes group, a notable enhancement was observed, as the CFU counts in the latter were 2.17 × 10^6^ CFU/g, resulting in a 68% decrease in fungal burdens with the addition of CFW-PEc. Furthermore, the CFW-PEc-(0.1 µM)-AmB-ethosomes group also showed a significant reduction to 1.92 × 10^6^ CFU/g, reflecting a 3.8-fold decrease. These results indicate that the incorporation of CFW-PEc significantly enhances the antifungal efficacy of AmB-loaded ethosomes.

### Histopathological evaluation of therapeutic efficacy in CFW-PEc-AmB-ethosomes treatment

**Fig 6:**
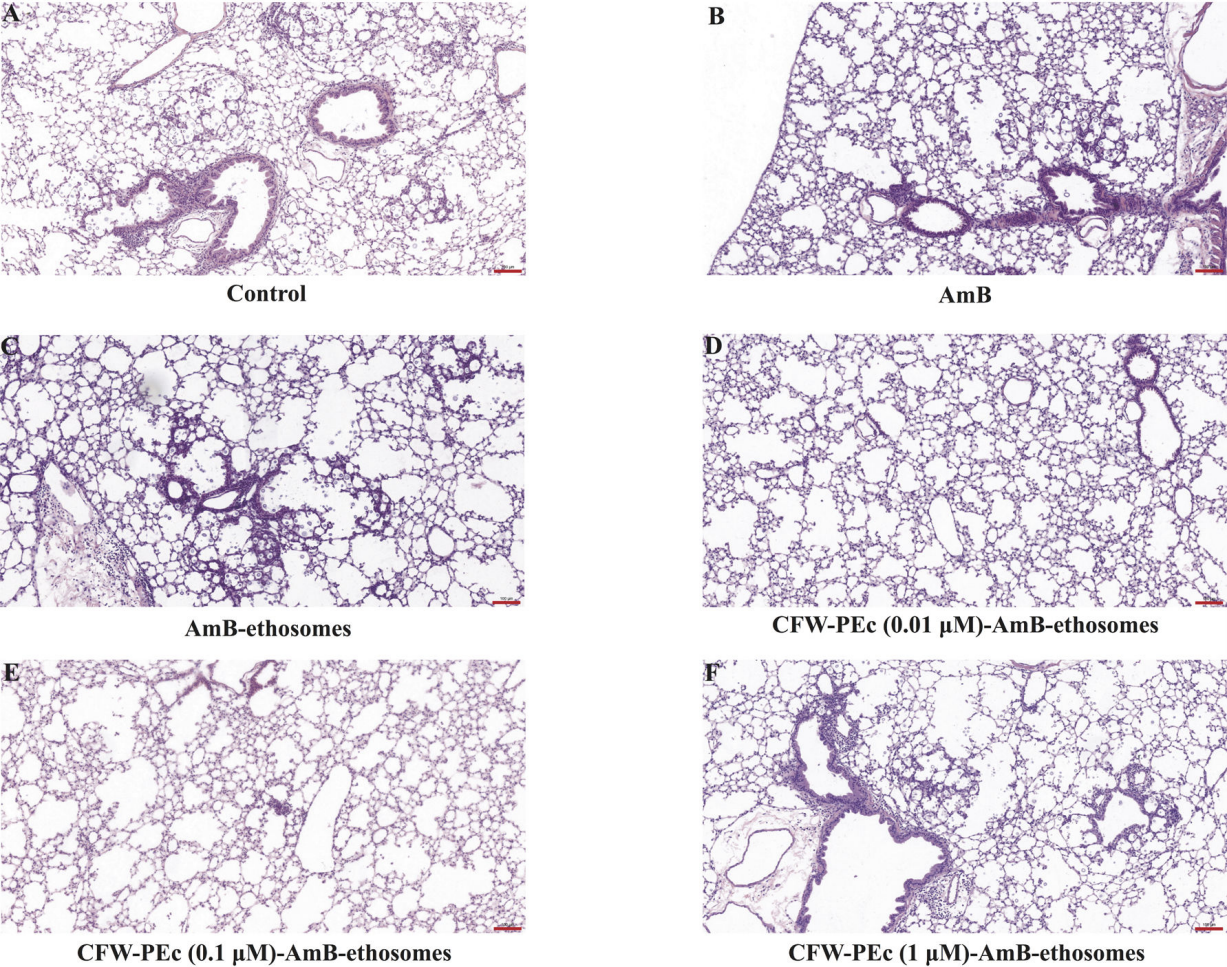
Histopathological evaluation of lung tissues treated with CFW-PEc-AmB-ethosomes. H&E-stained lung sections (**A–F**) show varied degrees of inflammatory cell infiltration across groups. The control group (**A**) exhibits extensive inflammation, while treatment with AmB (**B**) and AmB-ethosomes (**C**) shows moderate improvement. Notably, the CFW-PEc (0.01 µM)-AmB-ethosomes group (**D**) reflects minimal inflammatory response, correlating with decreased fungal burden, while higher concentrations of CFW-PEc demonstrate varying degrees of inflammation (**E and F**). All images include scale bars of 200 µm.

To further evaluate the therapeutic efficacy of these treatments, histopathological analysis was conducted across six groups, with lung sections stained using H&E, as illustrated in Fig. 6A through F. In the control group (Fig. 6A), significant infiltration of inflammatory cells was observed in the interstitium surrounding the alveoli, along with marked thickening and widening of the alveolar walls, indicating substantial pulmonary damage due to infection. In the groups treated with AmB (Fig. 6B), the alveolar walls exhibited notable widening, likely attributable to congestion in the pulmonary capillaries, while only minimal inflammatory cell infiltration was present. Conversely, the groups treated with AmB-ethosomes (Fig. 6C) showed that most alveolar walls appeared normal; however, some areas exhibited pulmonary interstitial lesions surrounded by considerable infiltration of inflammatory cells, reflecting ongoing inflammation. The introduction of CFW-PEc in the AmB-loaded ethosomes yielded even more promising histopathological results. The CFW-PEc-(0.01 µM)-AmB-ethosomes group (Fig. 6D) showed minimal inflammatory cell infiltration in a few alveoli, with the overall structure of the alveolar walls remaining largely intact. This finding is particularly noteworthy as it correlates with the substantial reduction in CFU counts observed in this treatment group. Similarly, the CFW-PEc-(0.1 µM)-AmB-ethosomes group (Fig. 6E) displayed minimal overall inflammatory cell infiltration, although certain regions still presented pulmonary interstitial lesions with extensive inflammatory cell presence. In contrast, the groups treated with CFW-PEc (1 µM)-AmB-ethosomes (Fig. 6F) exhibited patchy infiltration of inflammatory cells in some areas, indicating that the efficacy of the formulation may vary with dosage.

These results collectively highlight the dual role of CFW-PEc in not only enhancing the antifungal efficacy of AmB-loaded ethosomes, as evidenced by reduced fungal burdens, but also in mitigating the associated histopathological damage typically caused by *C. neoformans*. The preservation of alveolar architecture and the reduction of inflammatory responses in treated groups, particularly with lower amounts of CFW-PEc, suggest that this additive may enhance both the therapeutic effectiveness and safety profile of antifungal treatments within lung tissue.

## DISCUSSION

The maintenance of a uniform spherical shape is particularly significant, as it is associated with enhanced cellular uptake and improved pharmacokinetic profiles *in vivo*. Spherical nanoparticles tend to exhibit better stability and biodistribution properties, contributing to more effective drug delivery systems (38, 39). For instance, studies have demonstrated that spherical nanoparticles can facilitate the passive targeting of tumors through the enhanced permeability and retention effect, thus potentially increasing therapeutic efficacy (40, 41). Therefore, the promising morphological characteristics of the CFW-PEc-AmB-ethosomes suggest their potential suitability for further development as effective delivery systems in clinical applications. As an effective strategy in antifungal drug delivery systems, nanoparticles are pivotal in treating deep fungal infections due to their ability to enhance drug permeability across biological membranes (42). Key characteristics, such as particle size and zeta potential, are essential factors that influence their interactions with biological cells, as well as their capacity to facilitate drug release and improve cellular uptake efficiency (43, 44). A negative zeta potential typically suggests good nanoparticle stability, as higher negative values can enhance repulsion between particles, minimizing aggregation (45, 46). The consistent zeta potential values across the different formulations indicate that incorporating CFW-PEc does not significantly alter the surface charge of AmB-loaded ethosomes. Entrapment efficiency and drug loading improvements in CFW-PEc-AmB-ethosomes compared to AmB-ethosomes alone indicate a more effective incorporation of the antifungal agent within the delivery system. Higher drug loading enhances the therapeutic potential by delivering more drug to the target site (47). Additionally, the excellent storage stability observed over 2 weeks suggests that these formulations are viable for practical use, maintaining their structural and functional integrity over time. This stability may enhance interactions with biological cells, potentially facilitating cellular uptake and affecting the biodistribution and efficacy of the antifungal treatment (48).

The antifungal activity results reveal that CFW-PEc (0.01–1 µM)-AmB-ethosomes exhibit enhanced inhibition against *C. neoformans* cells compared to AmB-ethosomes alone. The consistently high inhibition rates of CFW-PEc (0.01 µM)-AmB-ethosomes across a broad range of concentrations suggest that the incorporation of CFW-PEc significantly improves the efficacy of AmB delivery. This enhancement is likely due to the synergistic effect of CFW-PEc’s inherent antifungal properties combined with the optimized drug delivery mechanism provided by ethosomes. The antifungal activity of CFW-PEc, evidenced by consistent inhibition rates between 78% and 80% across various concentrations, underscores its effectiveness against *C. neoformans*. CFW’s mechanism of binding to chitin chains in the fungal cell wall likely contributes to its sustained antifungal efficacy, providing a stable inhibitory effect when used in combination with AmB-ethosomes (49). This sustained activity is likely due to CFW’s ability to bind to chitin chains in the fungal cell wall, which enhances overall antifungal efficacy when combined with AmB-ethosomes (29). This synergistic effect clarifies the enhanced potency of CFW-PEc (0.01–1 µM)-AmB-ethosomes, as they not only capitalize on CFW-PEc’s inherent antifungal properties but also optimize drug delivery mechanisms. As a result, incorporating CFW-PEc into AmB-ethosomes markedly boosts antifungal activity, particularly at lower concentrations. Cytotoxicity assessments reveal that CFW-PEc exhibits a favorable safety profile *in vitro*, with higher cell viability observed at lower concentrations. When combined with AmB-ethosomes, CFW-PEc further enhances cell viability, particularly at the lowest concentration tested (0.01 µM), suggesting a protective effect on mammalian cells. However, higher concentrations of CFW-PEc (0.1 µM and 1 µM) result in reduced cell viability, highlighting the importance of optimizing the concentration to balance antifungal efficacy and cytotoxicity.

The photoluminescence of C6 is commonly employed to investigate the cellular absorption of non-fluorescent drug compounds using fluorescence microscopy (50–52). In this context, we investigated the impact of CFW-PEc on the effectiveness of nanoparticle drug delivery by quantitatively assessing the intracellular uptake of CFW-PEc (0.01–1 µM)-C6-ethosomes and C6-ethosomes in *C. neoformans* cells using confocal microscopy. The peak fluorescence observed at 6 hours indicates that CFW-PEc enhances the intracellular uptake of C6-ethosomes in a concentration-dependent manner, with 0.01 µM showing particularly effective cellular uptake. The observed blue fluorescence outside the cell walls indicates that CFW-PEc is binding to the chitin present on the outer surface, suggesting that this material may not be able to enter the cells. This binding to chitin on the fungal cell surface potentially limits its entry into cells at higher concentrations, creating a barrier that hinders the internalization of ethosomes and explains the reduced uptake observed with higher CFW-PEc concentrations. The substantially lower uptake of CFW-PEc (0.1 µM)-C6-ethosomes and CFW-PEc (1 µM)-C6-ethosomes highlights that increased amounts may hinder effective cellular penetration. This is potentially due to interactions between CFW-PEc and the chitin and chitosan present in the cell wall of *C. neoformans*. Importantly, the cell wall of *C. neoformans* contains a higher concentration of these polysaccharides compared to other fungi (53, 54), which may influence how varying amounts of CFW-PEc on ethosomes interact with them. As the amount of CFW-PEc increases, the binding affinity to chitin may also strengthen, potentially impacting the effectiveness of ethosome uptake. Elevated amounts could lead to increased binding but reduced availability for internalization, highlighting the importance of optimizing CFW-PEc levels on ethosomes for efficient drug delivery. The similarity between C6-ethosomes and CFW-PEc (0.01 µM)-C6-ethosomes in fluorescence patterns suggests that low concentrations of CFW-PEc maintain effective delivery while potentially enhancing targeting specificity. After binding to the cell wall, the ethosomes may undergo several potential mechanisms for drug delivery. One possibility is fusion with the fungal cell membrane, allowing direct release of encapsulated cargo into the cytoplasm, similar to how amphiphilic micelles transport across epithelial cells (50, 55). Alternatively, the ethosomes could be internalized via endocytosis-like processes, facilitated by the flexible lipid bilayer of ethosomes interacting with the dynamic cell wall components (56, 57). Evidence from our fluorescence microscopy indicates effective uptake at optimal CFW-PEc concentrations, with reduced internalization at higher levels, potentially due to excessive binding to chitin. While direct evidence in *C. neoformans* is limited, these mechanisms are supported by studies on nanoparticle-fungal interactions, and further experiments could provide confirmatory results. Therefore, optimizing CFW-PEc concentration is crucial for maximizing drug delivery efficiency while considering the unique composition of the *C. neoformans* cell wall.

The *in vivo* antifungal efficacy results further validate the enhanced therapeutic outcomes of CFW-PEc-AmB-ethosomes. The significant enhancement in the efficacy of CFW-PEc-(0.01 µM)-AmB-ethosomes against cryptococcal pneumonia can be attributed primarily to the targeting capability of CFW-PEc toward *C. neoformans*. The study results demonstrate that the inclusion of CFW-PEc in the AmB-loaded ethosomes leads to a striking reduction in fungal burdens in the lung tissues of infected mice, achieving a remarkable 10-fold decrease in CFU counts compared to the control group. This marked improvement underscores the importance of targeted delivery systems in antifungal therapy. The targeting function of CFW-PEc plays a crucial role in improving the drug’s therapeutic efficacy. By binding to chitin within the cell walls of *C. neoformans*, CFW-PEc enhances the localization of the drug directly at the site of infection. This interaction helps the fungal cells take up more of the drug, which weakens the structure of the fungal cell wall. As a result, the fungi become more vulnerable to the antifungal effects of AmB. The unique chitin-rich composition of *C. neoformans* provides an ideal platform for implementing such targeted strategies. In contrast to conventional AmB-loaded nanoparticles (58–60), the CFW-PEc formulation offers significant advantages. While AmB-ethosomes provide a general delivery system, the addition of CFW-PEc ensures a more precise targeting mechanism that enhances drug localization and effectiveness. This targeted action by CFW-PEc minimizes drug dilution in non-target tissues and optimizes the therapeutic impact at the infection site. These results collectively highlight the dual role of CFW-PEc in not only enhancing the antifungal efficacy of AmB-loaded ethosomes, as evidenced by reduced fungal burdens, but also in mitigating the associated histopathological damage typically caused by *C. neoformans*. The preservation of alveolar architecture and reduction in inflammatory responses in treated groups, particularly with lower amounts of CFW-PEc, suggest that this additive may enhance both the therapeutic effectiveness and safety profile of antifungal treatments within lung tissue. Moreover, the ability of CFW-PEc to leverage natural binding affinities ensures that the antifungal agent is concentrated where it is most effective, leading to improved outcomes in the treatment of cryptococcal pneumonia.

In conclusion, the findings from both CFU analyses and histopathological evaluations suggest that CFW-PEc is a valuable component in advancing treatment strategies for cryptococcal pneumonia. By demonstrating improved antifungal efficacy and reduced tissue damage, CFW-PEc reinforces the significance of targeted delivery systems in antifungal therapy. These findings emphasize the potential of CFW-PEc as an effective nanomaterial in enhancing antifungal therapy, particularly in improving the treatment of cryptococcal pneumonia and possibly other fungal infections. Future research should focus on refining these formulations and further exploring the underlying mechanisms of this targeting strategy to maximize therapeutic benefits.

## Data Availability

The authors confirm that the data supporting the findings of this study are available within the article.
